# Improving Acute Care Preparedness Amongst Medical Students: A Systematic Review

**DOI:** 10.7759/cureus.86688

**Published:** 2025-06-24

**Authors:** Haider Merchant, Joanna Tarr

**Affiliations:** 1 ENT, Royal Cornwall Hospital, Truro, GBR; 2 General Medicine, University of Exeter, Exeter, GBR

**Keywords:** acute care, acute medicine, a systematic review, emergency medicine, undergraduate medical education

## Abstract

Evidence suggests that there is inadequate preparation for acute care within the undergraduate medical curriculum. Although previous attempts have been made to address this concern, a lack of formal evaluation of intervention effectiveness limits their utility.

This review aimed to identify educational interventions seeking to prepare medical students for acute care and evaluate their effectiveness.

MEDLINE, CENTRAL, Embase, Scopus and Web of Science were systematically searched. Primary research studies published between 2000 and 2020 and reporting changes in outcomes related to medical student preparation for acute care were included. Study outcomes were described as either highly effective, effective, ineffective, negative or variable. Study quality was appraised using the Medical Education Research Study Quality Instrument (MERSQI). Studies with an MERSQI score of ≥14 were classed as high-quality.

Overall, 72 studies were included in this review. The majority were single-group pre- and post-test studies (n=39, 54.2%) and none measured changes in student behaviour or patient/healthcare outcomes. Courses, clerkships, and simulation were found to be the most effective interventions. All Clerkship studies measuring improvements in acute care skills were effective or highly effective. Mean MERSQI score was 12.4 (range=7.8-15.5, SD=1.7) and 18 studies (25%) were classed as high-quality.

This review favours the use of clerkships, as well as courses and simulation. However, considerable heterogeneity and numerous study limitations prevent firm conclusions from being drawn. Future high-quality studies, especially those measuring behavioural changes and patient/healthcare outcomes, are subsequently needed. Reviews with a more focused area of research, those assessing long-term outcomes and cost-effectiveness, would additionally prove beneficial.

## Introduction and background

Background

The World Health Organisation (WHO) defines acute care as the treatment of “sudden, often unexpected, urgent or emergent episodes of injury and illness that can lead to death or disability without rapid intervention”[1]. This encompasses the following areas within medicine: emergency medicine, trauma care and acute care surgery, critical care and pre-hospital care. Although the General Medical Council’s Outcomes for Graduates 2018 document states that, “Newly qualified doctors must be able to give immediate care to adults, children and young people in medical and psychiatric emergencies,” preparedness for acute care has been recognised as a particular area of concern [2].

The problem

A large retrospective study by Jen et al. identified higher patient mortality rates for emergency admissions on the day that the new foundation doctors (newly qualified doctors in the United Kingdom) started work compared with the previous week [3]. A systematic review by Tallentire et al. identified that UK graduates felt least well prepared in delivering acute care, when compared against other outcomes in Tomorrow’s Doctors 2009 and that preparedness in acute care was declining [4,5]. Two more recent reviews, in addition to a preparedness to practice survey, have also found acute care to be a predominant area in which newly qualified doctors feel unprepared [6-8].

These findings strongly suggest that the undergraduate curriculum is not sufficiently preparing medical graduates for acute care responsibilities. Previous reviews have attempted to address this concern [9,10]. In particular, the review by Smith et al., which characterised the problem of suboptimal training amongst undergraduates in the care of the acutely ill patient and identified potential educational interventions [11]. However, a formal assessment of intervention effectiveness was not undertaken in these reviews. Consequently, there is a lack of clarity as to whether the interventions described would succeed, and which may prove the most successful. This limitation, as well as the subsequent publication of the studies cited above, indicates that uncertainty still remains with regard to improving the provision of acute care education at the undergraduate level.

Rationale for the review

A review evaluating the effectiveness of previously employed educational approaches may address this uncertainty by providing an overview of which interventions are most likely to improve undergraduate preparedness for acute care. This information can be used by medical school faculty members and educational researchers to design and develop future curricula and educational interventions to address this concern. This will ultimately ensure that future newly qualified doctors are sufficiently prepared to care for acutely unwell patients.

Aims

A systematic review of the peer-reviewed literature was conducted in order to identify and describe previously published educational interventions seeking to prepare medical students for the assessment/management of acutely unwell patients and evaluate their effectiveness.

## Review

Methods

This systematic review was conducted and written in accordance with the Preferred Reporting Items for Systematic reviews and Meta-analyses (PRISMA) guidelines to ensure high methodological rigour [12]. Prior to its undertaking, a protocol was developed and registered on the PROSPERO registry (Reg No CRD42020178992).

Search Strategy

The search strategy was developed using Ovid MEDLINE and adapted for each database (Appendices: Table 5). Relevant keywords and indexing terms, such as medical subject headings for MEDLINE, were included and combined with the Boolean operators “And...OR.”

Initial search terms were sought from those present in the WHO definition of acute care [1]. A pilot search on MEDLINE and Embase was conducted to refine the search strategy.

Eligibility Criteria

Studies were required to meet an inclusion criterion. Studies were required to be published between 2000 and 2020, and published in the English language, or a suitable English translation was available. Only primary research studies, conducted on medical students or medical student data presented individually, were included. Quantitative study designs (e.g. randomised controlled trials), reporting pre- and post-intervention data (this was needed to determine intervention effectiveness), were sought for. Single-group post-test only, cross-sectional and controlled studies with post-test only data were excluded. Studies describing educational interventions aimed at preparing medical students for the assessment/management of acutely unwell patients were included. All modalities of educational interventions were included, provided their content corresponded with the WHO’s six domains of acute care and involved general or specific skills/responsibilities that were mapped against Outcomes for Graduates 2018 [1,2]. Studies were required to report changes in one or more outcomes pertaining to the assessment/management of acutely unwell patients, with respect to Kirkpatrick’s hierarchy of attitudes, knowledge, skills, behaviour and patient/healthcare outcomes [13].

Study Selection

MEDLINE, CENTRAL, Embase, Scopus and Web of Science were searched by HM. The identified records were uploaded onto the Rayyan QCRI software, which was used to remove duplicates and record decisions [14].

Two thousand one hundred eighty-one records were initially identified after searching the above databases [15]. After removal of duplicates (n=597), 1584 of the remaining titles and abstracts were screened against the eligibility criteria. HM and JT independently screened the first 9.5% (n=150) of titles and abstracts. Disagreements arose in 9.3% (n=14) of cases, which were resolved through discussion. HM screened the remainder of the titles and abstracts alone, consulting JT for advice where needed.

One hundred forty records (1444 excluded) remained following title-abstract screening. Full texts of these records were retrieved and re-assessed against the eligibility criteria. HM and JT independently assessed the first 10% (n=14) of these studies. There were no disagreements. HM assessed the remaining articles alone, consulting JT for advice where needed. Of these full-text papers assessed, 60 were included in this review.

**Figure 1:**
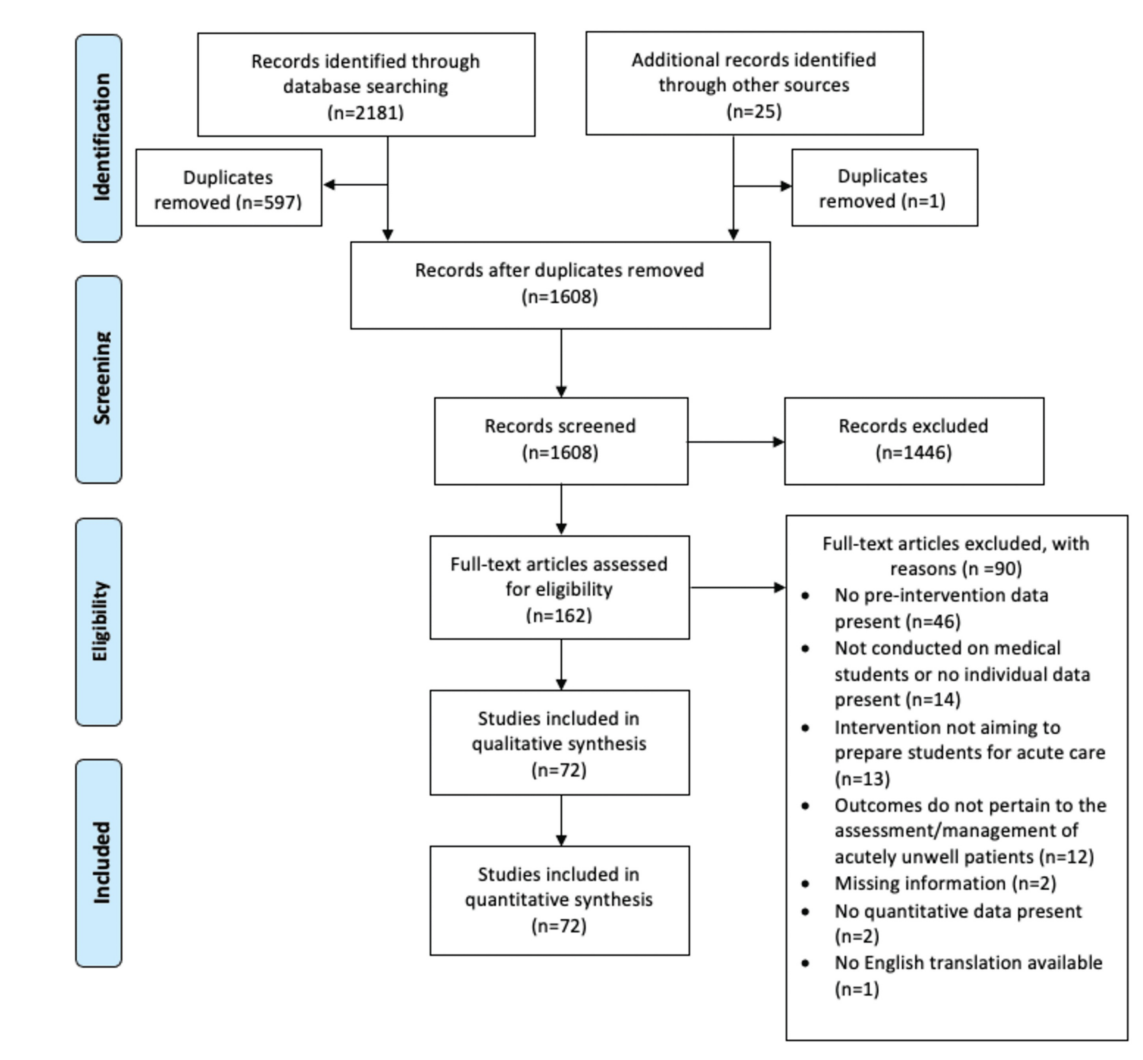
PRISMA flow diagram of search results

HM then hand-searched the reference lists of the included papers, which yielded 25 records. After the removal of one duplicate, two were excluded at the title/abstract stage and 10 after full-text assessment. This yielded 12 additional studies. Figure 1 presents a PRISMA flow diagram of the overall search results (including those identified from hand-searching the reference lists) and the reasons for exclusion.

Data Extraction

HM independently extracted and coded the data onto a standardised data extraction form, which was developed using items from the Medical Education Research Study Quality instrument (MERSQI) and guidance from chapter five of the Cochrane Handbook for Systematic Reviews of Interventions [11,16]. Four studies were found to have missing data. HM contacted the corresponding authors by email, of whom two provided the missing data. The remainder were excluded under “missing information” as stated in Figure 1.

The information sought from each study initially included their study characteristics (authors, publication year, study design). Second, their population demographics (country of origin, student year of study, number of participants). Third, the topic area within acute care and the description of the educational intervention. Fourth, the outcomes measured and the measurement methods. Finally, the pre- and post-test results with respective p-values and confidence intervals (CIs) if reported.

Synthesis

Considerable heterogeneity among the included studies precluded meta-analysis. Therefore, the findings of this review were conveyed narratively.

Intervention Types

Conventional content analysis of the study titles and text descriptions of each intervention was undertaken to categorise the interventions by type [17]. The categorisation system was initially developed by HM and modified after discussions with JT. Appropriate subtypes were also developed where needed.

Intervention Effectiveness

The results that corresponded to the highest measured outcome-level of each study (attitudes< knowledge< skills< behavioural changes< patient/healthcare outcomes), which was based on a combination of Kirkpatrick’s and Miller’s hierarchy, were used to determine intervention effectiveness [13,18].

A modified version of a framework previously described by Brennan and Mattick, which was adapted from Gill et al. and achieved an interrater reliability of >95% agreement, was employed [19,20]. Each study was subsequently allocated an effect measure, as depicted in Table 1. 

**Table 1:** The effect measures and their descriptors

Effect-Measure	Criteria
Highly effective (++)	Pre and post-test studies: The intervention produced a >50% change in the positive direction from baseline in the majority of outcomes measured at the first post-measure. Studies with control/comparator groups: The absolute change in the positive direction from pre to post-test results in the intervention group >50% higher versus the control/comparator group in the majority of outcomes measured at the first post-measure.
Effective (+)	Pre and post-test studies: The intervention produced a 20-50% change in the positive direction from baseline in the majority of outcomes measured at the first post-measure. Studies with control/comparator groups: The absolute change in the positive direction from pre to post-test results in the intervention group was 20-50% higher versus the control/comparator group in the majority of outcomes measured at the first post-measure. If equal numbers of outcomes are + and ++ then overall is +.
Ineffective (O)	The intervention produced a <20% change in the positive direction from baseline, or there was a <20% difference between the intervention and control/comparator group changes.
Negative (-)	The intervention produced a change in the reverse direction, or the control/comparator group produced a ≥20% change in the positive direction versus the intervention group.
Variable (V)	Includes both a positive (+ or ++) and a negative (-) or ineffective (O) outcome.

Quality Assessment

Study quality was appraised using the MERSQI as it is a reliable and valid means of appraising quantitative studies [15,21]. The MERSQI evaluates studies based on six domains (study design, sampling, type of data, validity of the evaluation instrument, data analysis and outcomes), each of which is scored on a three-point scale, giving a total score out of 18 for each study [22]. As in previous reviews, MERSQI scores were calculated as a percentage of the total achievable points (some items were non-applicable) and then multiplied by 18 to produce a total score out of 18. Studies with an MERSQI score of ≥14 were defined as high-quality, as this has been conducted in previous reviews [23-26].

Results

Study Characteristics

Overall, 72 studies were included in this review [27-98]. The key findings of each study are presented in Table 2.

**Table 2:** Key findings of each study ACS: acute coronary syndrome; AED: automated external defibrillator; ALS: advanced life-support; ALSO: advanced life-support in obstetrics; ATLS: advanced trauma life-support; BLS: basic life-support; CCM: critical care medicine; CBD: case-based discussion; CPR: cardiopulmonary resuscitation; CRM: crew resource management; EM: emergency medicine; EMS: emergency medical services; HFMS: high-fidelity mannikin simulation; LFS: low-fidelity simulation; MCQ: multiple-choice questions; OSCE: observed structured clinical examination; PBL: problem-based learning; PBLS: paediatric basic life-support; RAPID: Resuscitation, Analgesia and assessment, Patient needs, Interventions, Disposition; RCT: randomised controlled trial; RTP: resident as teacher preceptorship; SAGT: situation awareness global assessment tool; SAQ: short-answer questions; STP: stop the bleed; TEAM: Trauma Evaluation and Management; VP: virtual patient; OSCE: Observed Structured Clinical Examination

Study	Population		Intervention				Methods		Results		
	Country of origin	Participant number (n)	Study design	Topic area within acute care	Intervention type/details	Control/comparator group details	Outcome(s) assessed	Measurement method(s)	Change in pre- vs. post-test outcomes	Effect- measure (++, +, O, - or V)	MERSQI score (/18)
Schroll et al. (2020) [27]	USA	443	Pre and post-test	Haemorrhage control	STB course	n/a	Confidence in haemorrhage management	Mean five-point Likert-scale questionnaire score	2.5 to 4.7	++	8.4
Smith et al. (2019) [28]	USA	65	RCT	General emergency care	RTP clerkship	Standard clerkship	Emergency management skills	Mean five-point Likert-scale global score OSCE	Increase by 1.2 in intervention group vs 0.9 in control group (p=0.167)	+	14.5
Schroll et al. (2019) [29]	USA	423	Pre and post-test	Haemorrhage control	STB course	n/a	1. self-reported knowledge acquisition, 2. confidence in haemorrhage management	1. Mean percentage of students, 2. Mean five-point Likert-scale questionnaire score	1. 17.9% to 89.3% (p<0.0001) 2. 2.5 to 4.7 (p<0.0001)	++	10.8
Lei et al. (2019) [30]	USA	123	Pre and post-test	Haemorrhage control	STB course	n/a	Haemorrhage management knowledge	MCQ-test pass rates (%)	73% to 100%	+	12
Berger et al. (2019) [31]	Germany	127	RCT	Resuscitation	HFMS + PBL	Standard hands-on CPR training	Self-perception of CPR skills	Mean six-point Likert-scale questionnaire score	Increase by 1.4 in intervention group vs 1.3 in control group (p=0.9)	O	12
Padaki et al. (2018) [32]	USA	18	Pre and post-test	In-flight medical emergencies	LFS	n/a	Knowledge on the management of in-flight emergencies	Mean MCQ-score (/15)	11.3 to 13.1 (p=0.001, CI=0.87,2.57)	O	12
Hill et al. (2018) [33]	Kenya	61	Pre and post-test	Trauma	TEAM course with LFS	n/a	Trauma management knowledge	Mean MCQ-score (/20)	11.4 to 14.4 (p<0.001)	+	10
Egro et al. (2018) [34]	USA and UK	79	RCT	Acute burns management	E-learning	Lecture-based learning	Knowledge on acute burns management	Mean closed written-test score (% points)	Increase by 50.2% in intervention group vs 44.8% in control group (p>0.05)	O	14.5
Abbas et al. (2018) [35]	Syria	72	RCT	Resuscitation	Peer-led BLS course	Professional-led training course	BLS knowledge	Mean MCQ-score (/60)	Increase by 15.9 (p=0.08) in professional-led group vs 14.3 in peer-led group (p=0.9)	O	14.5
Kwan et al. (2017) [36]	USA	43	Pre and post-test	Cardiac emergencies	HFMS	n/a	Self-reported competence in: 1. identification of ACS, 2. identification of cardiac arrhythmias, 3. Management of ACS, 4. Management of cardiac arrhythmias	Mean five-point Likert-scale questionnaire score	1. 3.5 to 4.4 2. 3 to 4.4 3. 3.3 to 4.4 4. 2.6 to 4.1	+	7.8
House et al. (2017) [37]	USA	176	Non-randomised two-group	Cardiac emergencies	Peer-led simulation	Professional-led simulation	Knowledge on assessment and management of cardiac emergencies	Mean written-test score (% points)	Increase by 9% in intervention group vs 10% in control group (p=0.6)	O	10.5
Stroben et al. (2016) [38]	Germany	30	Pre and post-test	General emergency care	Simulated live patients	n/a	Self-reported preparedness	Mean -3 to +3 Likert-scale questionnaire score	-0.3 to +0.7 (p<0.001)	++	9.6
Sánchez-Ledesma et al. (2016) [39]	Spain	300	Pre and post-test	Neurological emergencies	HFMS	n/a	Simulated neurological emergency assessment and management skills	Rates of demonstration of 14 specific competencies	>50% increased demonstration of all competencies (p<0.05 for all but two)	++	11
Reed et al. (2016) [40]	USA	135	Pre and post-test	Emergency skills	LFS + E-learning	n/a	Simulated emergency management skills	Mean overall checklist score (/100)	54.6 to 99.3 (p<0.05)	++	13
MacEwen et al. (2016) [41]	UK	205	Pre and post-test	Diabetic emergencies	“Diabetes acute care day” course	n/a	Knowledge on diabetic emergency management	Mean MCQ-score (/10)	2.7 to 4.7 (p<0.001)	++	11
Jordan et al. (2016) [42]	USA	53	Non-randomised two-group	General emergency care	Curriculum modification (CBD during clerkship)	Self-directed learning	EM knowledge	Mean MCQ-score (/15)	Increase by 2.9 in intervention group vs 0.7 in control group (p<0.001)	++	14.5
Hogg and Miller (2016) [43]	UK	165	Pre and post-test	General emergency care	Simulated live patients	n/a	Confidence in the identification and management of life-threatening emergencies	Mean 5-point Likert-scale questionnaire score	3.4 to 4.6 (p<0.01)	+	10.2
Delgado-Reyes et al. (2016) [44]	Mexico	115	Pre and post-test	Trauma	TEAM course	n/a	Trauma management knowledge	Mean MCQ-score (/10)	5 to 7.3 (p<0.01)	+	12
Williams et al. (2015) [45]	UK	24	Pre and post-test	Cardiac emergencies	Simulated live patients	n/a	Knowledge on management	Mean SAQ-score (/43)	25 to 34 (p<0.001)	+	11.5
Treadwell (2015) [46]	South Africa	82	Pre and post-test	Emergency skills	LFS	n/a	EM skills	Mean OSCE checklist score (% points)	19.3% 69% (p=0.0001)	++	12
Solymos et al. (2015) [47]	Ireland	41	RCT	Sepsis	HFMS	Didactic lecture-based teaching	Knowledge on the recognition and management of Sepsis	Mean MCQ-score (/25)	Increase by 6.8 in intervention group vs 4.5 in control group (p=0.0387)	++	12.5
Ruest et al. (2015) [48]	USA	55	RCT	General emergency care	RTP clerkship	Standard clerkship	Simulated emergency management skills	Mean Five-point Likert-scale global performance score	Increase by 1 in intervention group vs 0.5 in control group (p=0.026)	++	12.5
Rivkind et al. (2015) [49]	Israel	490	Pre and post-test	Trauma	Course with simulated live patients	n/a	Trauma management knowledge	Mean MCQ-score (% points)	58.2% to 67.9% (p<0.001)	O	13
Pean et al. (2015) [50]	Haiti	115	Pre and post-test	Emergency skills	Peer-led emergency skills course	n/a	emergency skills knowledge	Mean MCQ-score (% points)	36% to 68% (p<0.001)	++	12
Mughal et al. (2015) [51]	UK	66	Pre and post-test	Acute surgical care	Course with HFMS	n/a	knowledge on surgical emergencies	Mean MCQ-score (% points)	57.9% to 70.9% (p<0.0001)	+	12.5
Lehmann et al. (2015) [52]	Germany	57	RCT	Paediatric resuscitation	Blended learning using VPs (E-learning)	Standard PBLS training	observed PBLS performance	Mean: 1. Overall checklist score (% points), 2. Total time(s) of PBLS	1. Increase by 27.2% in control group vs 14.6% in intervention group 2. Decrease by 12.5s in control group (p=0.05) vs 10s in intervention group (p<0.001)	-	15.5
Cuisinier et al. (2015) [53]	France	19	Pre and post-test	Trauma	EM course with HFMS	n/a	Simulated trauma assessment and management skills	Evaluation of three skillsets (median checklist scores): 1. admission, equipment and patient safety (/18) 2. systematic clinical examination (/27) 3. principal injury diagnosis and therapy (/15)	1. 10 to 13 (p<0.01) 2. 8 to 12 (p<0.01) 3. 7 to 9 (p<0.02)	+	12
Couto et al. (2015) [54]	Brazil	163	Non-randomised two-group	Paediatric emergencies	HFMS	CBD	Knowledge on the assessment and management of: 1. Anaphylaxis, 2. Supraventricular tachycardia	Mean MCQ-score (% points)	1. Increase by 21.2% in control group vs 19.9% in intervention group (p<0.05) 2. Increase by 16.1% in control group vs 8.6% in intervention group (p<0.05)	-	14
Cortegiani et al. (2015) [55]	Italy	98	RCT	Resuscitation	HFMS + didactic teaching	Didactic-only teaching	ALS knowledge	Mean true-false test score (/100)	Increase by 40.3 in intervention group vs 35.3 in control group	O	13.5
Woods et al. (2014) [56]	Canada	42	Non-randomised two-group	General emergency care	Clerkship with RAPID	Standard	Verbalised assessment and management of an acutely unwell patient	Mean case checklist score (/16)	Increase by 1.9 in intervention group vs 0.3 in control group (p=0.006)	++	11.5
Woodfield et al. (2014) [57]	UK	35	Pre and post-test	Acute prescribing	LFS	n/a	drug-chart safety for medical emergencies	Median number of drug errors	59 to 9 (p<0.05)	++	11
Langdorf et al. (2014) [58]	USA	19	Pre and post-test	Resuscitation	HFMS	n/a	Simulated cardiac arrest scenario performance	1. Mean time(s) to first CPR, 2. Mean time (min) to first defibrillation, 3. mean total scenario score (/121), 4. No. of students achieving return of spontaneous circulation, 5. Identification rate of myocardial infarction	1. 113s to 13s (p=0.004), 2. 3 min to 1.5 min (p=0.03), 3. 45 to 98 (p<0.0001), 4. 3 to 14 (p=0.001), 5. 100% increase	++	13
Anđelić et al. (2014) [59]	Serbia	437	Pre and post-test	Resuscitation	Course with HFMS	n/a	Resuscitation knowledge	Mean SAQ-score (/25)	21.5 to 24.5	O	12
Manikam et al. (2013) [60]	UK	179	RCT	Paediatric emergencies	E-learning	Online dummy package	Knowledge on acute breathing difficulties	mean written knowledge-test score (/24)	Increase by 6.8 in intervention group (CI=5.56-8.12, p<0.05) vs 0.43 in control group (CI=-1.26-2.13, P>0.05)	++	14
Jordan et al. (2013) [61]	USA	48	Non-randomised two-group	General emergency care	E-learning	Didactic lecture-based teaching	Acute care knowledge	Mean MCQ-score (% points)	Increase by 28.4% in control group vs 9.9% in intervention group (p=0.0001, CI=10.40 to 26.50)	-	13.5
Homaifar et al. (2013) [62]	Rwanda	67	Pre and post-test	Acute obstetrics	ALSO course	n/a	Knowledge on obstetric emergencies	Mean SAQ-test score (/100)	54 to 74.6	+	12.5
Freund et al. (2013) [63]	France	310	Pre and post-test	Resuscitation	HFMS	n/a	Confidence in performing BLS	No. of students feeling confident on questionnaire	64 to 214 (p<0.001)	++	10.2
Ahmadi et al. (2013) [64]	Iran	24	Pre and post-test	Trauma	ATLS course (includes multiple simulation types)	n/a	Simulated trauma management scenario performance	Three OSCE domains (mean checklist scores): 1. knowledge of diagnostic and therapeutic procedures, 2. sequence of procedures, 3. skill performance	1. -38.5 to +27.2 (p<0.001) 2. 13.5 to 47.3 (p=0.016) 3. 15.5 to 58 (p=0.01)	++	11
Kononowicz et al. (2012) [65]	Poland	226	RCT	Resuscitation	VP E-learning module	Students not using VPs, control group (no access to VPs)	BLS-AED knowledge	Mean true/false test score (/60)	Increase by 11.4 in VP group (p<0.001) vs 8.7 in the no VP group (p<0.001) and 8.9 in the control group (p<0.001)	+	14
Hansel et al. (2012) [66]	Germany	61	RCT	Sepsis	CRM course	Simulation training, control group (no training)	1. Situational awareness in a simulated sepsis scenario, 2. scenario performance	1. Mean SAGT questionnaire score (/13), 2. Mean scenario checklist score (/15)	1. Increase by 1.3 in the simulation (p=0.04) and control groups (p=0.06) vs 1 in CRM group (p=0.14), 2. Increase by 1.6 in CRM group vs 0.1 in the Sim (p>0.99) and control groups (p=0.75)	V	15.5
Thomson et al. (2011) [67]	Australia	21	Pre and post-test	Paediatric resuscitation	LFS + E-learning	n/a	BLS and ALS knowledge	Median MCQ-score (/23)	12 to 21 (p<0.001)	++	12
Nicol et al. (2011) [68]	Australia	131	Pre and post-test	Resuscitation	Vertically integrated curriculum modification	n/a	BLS skills	Pass rate of students in a practical assessment (%)	36.3% to 64.3% (p<0.05)	++	12
Halm et al. (2011) [69]	USA	50	Pre and post-test	Toxicology	HFMS + PBL	n/a	Toxicology knowledge	Mean MCQ-score (% points)	59% to 80% (p<0.05)	+	11
Afzalimoghaddam et al. (2011) [70]	Iran	98	Pre and post-test	General emergency care	EM clerkship	n/a	EM knowledge	Mean MCQ-score (/50)	19.9 to 35.9 (p<0.0001)	++	13
Ten et al. (2010) [71]	USA	83	RCT	Acute surgical care	HFMS	CBD	Efficiency of assessment and management of a simulated patient with acute abdominal pain	Mean time(s) to complete the following tasks: 1. initiate cardiac monitor, 2. initiate blood pressure monitor, 3. order an intravenous line, 4. order an abdominal film, 5. order initial bloods mean no. of: 6. examination items found, 7. history items found, 8. students asking about allergies	1. decrease by 179.9s in control group vs 142s in intervention group, 2. decrease by 115.2s in control group vs 108.7s in intervention group, 3. decrease by 157s in intervention group vs 124.3 in control group, 4. decrease by 135.4 in intervention group vs 93.7 in control group, 5. decrease by 220.9s in intervention group vs 120.5 in control group, 6. decrease by 0.4 in intervention vs 0 in control group, 7. decrease by 0.7 in intervention group vs increase by 0.3 in control group, 8. increase by 3 in both groups	V	15.5
O’Leary and Janson (2010) [72]	Australia	28	Pre and post-test	Paediatric resuscitation	E-learning	n/a	Observed performance of: 1. BLS 2. ALS	No. of students who passed (n)	1. 8 to 21 (p<0.001, CI=34.9-80.5) 2. 0 to 21 (p<0.001, CI=61.8-99.8)	++	14
Merlin et al. (2010) [73]	USA	49	Pre and post-test	Resuscitation	EM clerkship	n/a	1. Self-perceived understanding of the triage system, 2. comfort in using a defibrillator	Mean four-point Likert-scale questionnaire score	1. 4.2 to 4.5 (p=0.00314) 2. 2.9 to 3.2 (p=0.04)	O	10.2
Nguyen et al. (2009) [74]	USA	63	Pre and post-test	Sepsis	Course with HFMS	n/a	Knowledge on: 1. early goal-directed therapy concepts, 2. assessment and management of severe sepsis/septic shock	1 and 2. Mean MCQ-score (% points)	1. 69.6% to 90.2% (p<0.05) 2. 40% to 76.5% (p<0.05)	+	12
Lin et al. (2009) [75]	Taiwan	94	Pre and post-test	Trauma	Emergency trauma training course	n/a	Confidence in managing trauma patients	Mean five-point Likert-scale questionnaire score	2 to 2.8 (p<0.001)	+	10.2
Carrero et al. (2009) [76]	Spain	70	RCT	Resuscitation	Curriculum modification with CBD	Traditional multimedia presentation	BLS knowledge	1. median SAQ-score (/3) 2. spotting errors on a BLS video, median score (/4)	1. increase by 1 in intervention group (p<0.01) vs 0.5 in control group (p>0.05), 2. increase by 1 in both intervention and control groups (p>0.05)	V	14.5
Ali et al. (2009) [77]	Canada	70	RCT	Trauma	TEAM course with HFMS and live patient simulation	Classroom-based TEAM course	Trauma management knowledge	Mean MCQ-score (% points)	Increase by 33.1% in the live patient group and by 35.2% in the mechanical model group vs 27% in the control group (p<0.05)	+	14.5
Vincent et al. (2008) [78]	USA	24	Pre and post-test	Trauma	Virtual reality simulation + E-learning	n/a	Simulated trauma scenario performance	1. triage score (/15), 2. Intervention score (/5), 3. Time to triage(min)	1. 9.7 to 14 (p<0.001) 2. 3.4 to 4.7 (p<0.001) 3. 8.2min to 4min (p<0.001)	+	11
Lampe et al. (2008) [79]	USA	42	Non-randomised two-group	General emergency care	EM sub-internship clerkship	Standard clerkship	EM knowledge	Mean MCQ-score (% points)	Increase was 13.4% higher for intervention group vs control (p=0.014)	O	14.5
Shukla et al. (2007) [80]	USA	240	Pre and post-test	Trauma	HFMS	n/a	Confidence in: 1. managing a major trauma patient, 2. working in a team to manage a major trauma patient	Five-point Likert-scale questionnaire	1. 1.9 to 3 (p<0.05) 2. 2.9 to 3.8 (p<0.05)	+	10.2
Breckwoldt et al. (2007) [81]	Germany	198	RCT	Resuscitation	Curriculum modification (BLS training at schools)	Standard BLS/ALS training, attendance with EMS	Resuscitation knowledge	Median SAQ-score (% points)	Increase by 11.8% in schools group vs 11.6% in EMS group, and 14.7% for standard training group	-	12.5
Ali et al. (2007) [82]	West Indies	70	RCT	Trauma	TEAM course with live patient simulation	Classroom-based TEAM course, control (no course)	Trauma management knowledge	Mean MCQ-score (% points)	Increase by 30.9% in new-TEAM course group vs 27.3% in old-TEAM course group (p=0.014), and 1% in the control group	V	14.5
Steadman et al. (2006) [83]	USA	34	RCT	General emergency care	HFMS	PBL	Acute care assessment and management skills	Mean overall checklist score (% points) across nine simulated cases	Increase by 24% in Simulation group vs 8% in PBL group (p<0.04)	++	13.5
Morgan et al. (2006) [84]	Canada	370	Pre and post-test	Cardiac emergencies	HFMS	n/a	Team simulated scenario performance	Mean overall checklist score (% points)	51.8% to 68.2% (p<0.0001)	+	14
MacDowall (2006) [85]	UK	23	Pre and post-test	General emergency care	HFMS	n/a	Self-reported confidence in assessment and management of an acutely ill patient	Mean five-point Likert-scale questionnaire score	2.9 to 3.7 (p<0.05)	+	10.2
Gordon et al. (2006) [86]	USA	38	RCT	General emergency care	HFMS	Didactic lecture-based learning	Acute care knowledge	Mean SAQ-score (/100)	Increase by 11.3 in lecture-group (CI=5.7- 16.9) vs 8.9 in Simulation group (CI=2.3-15.3) (p>0.05)	-	13.5
Cherry et al. (2005) [87]	USA	114	Non-randomised two-group	Trauma	TEAM course	No course	Trauma management knowledge	Mean MCQ-score (% points)	Increase by 7.8% in intervention group vs (p<0.05) vs a decrease by 4.5% in the control group (p>0.05)	++	13.5
Ali et al. (2005) [88]	Multiple	294	RCT	Trauma	TEAM course	No course	Trauma management knowledge	Mean MCQ-score (% points)	Increase by 20.7% (p<0.05) in intervention group vs a decrease by 2.4% in the control group	++	13.5
Weller et al. (2004) [89]	New Zealand	71	Pre and post-test	Acute surgical care	HFMS	n/a	Simulated acute care scenario performance	For three scenarios: 1. median global score (/5), 2. mean checklist score (/21, /20 and/21)	1. 2 to 3.2, 2.3 to 2.7 and 2.3 to 2.8 (p<0.001 for all) 2. 13 to 17 (p=0.01), 13 to 16 (p=0.04) and 14 to 15 (p=0.04)	+	11
Curran et al. (2004) [90]	Canada	50	RCT	Paediatric resuscitation	HFMS	Training video	Neonatal resuscitation confidence	Mean total Likert-scale questionnaire score (/75)	Increase by 18.6 in intervention group (p=0.000) vs 15.1 in the control group (p=0.000)	+	12
Ali et al. (2004) [91]	Australia	73	RCT	Trauma	TEAM course	No course	Trauma management knowledge	Mean MCQ-score (% points)	Increase by 17.1% in intervention group (p=0.0001) vs a decrease by 1.8% in the control group (p>0.05)	++	14.5
Ali et al. (2003) [92]	Jamaica	32	RCT	Trauma	TEAM course	No course	Trauma management knowledge	Mean MCQ-score (% points)	Increase by 16.3% in intervention group (p<0.001) vs a decrease by 3.1% in the control group (p=0.32)	++	13.5
Ali (2003) [93]	Canada	29	RCT	Trauma	TEAM course	No course	Trauma management knowledge	Mean MCQ-score (% points)	Increase by 34.4% in intervention group (p<0.05) vs a decrease by 3% in the control group	++	13.5
Morgan et al. (2002) [94]	Canada	144	RCT	General emergency care	HFMS	Video-based learning	Simulated recognition and management of the following emergencies: 1. myocardial ischaemia, 2. Anaphylaxis, 3. hypoxaemia	Mean checklist score (/12) for each scenario	1. Increase by 5.1 in control group vs 4.5 in intervention group (p=0.47), 2. increase by 5.2 in intervention group vs 3.9 in control group (p=0.09), 3. Increase by 1 in intervention group vs 0.9 in control group (p=0.92)	V	13.5
Ali et al. (2002) [95]	West Indies	32	Non-randomised two-group	Trauma	TEAM course	No course	Simulated trauma assessment and management skills	OSCE performance: 1. mean overall score (% points), 2. mean priority score (7-point scale), 3. mean approach score (5-point scale), 4. no. of students who passed	1. Increase by 28.3% in intervention group (p<0.05) vs 1.5% in the control group (p>0.05), 2. increase by 2.7 in intervention group (p<0.05) vs a decrease by 0.8 in the control group (p>0.05), 3. Increase by 2.5 in intervention group (p<0.05) vs a decrease by 0.3 in the control group (p>0.05), 4. Increase by 15 in intervention group (p<0.05) vs a decrease by 2 in the control (p>0.05)	++	12.5
Rogers et al. (2001) [96]	USA	26	Pre and post-test	General emergency care	CCM elective	n/a	Observed performance of the assessment and management of an acutely unwell patient	Mean: 1. overall OSCE score (% points), 2. computer simulator checklist score (% points)	1. 47% to 76% (p<0.0001) 2. 41 to 62% (p<0.0001)	++	12
Celenza et al. (2001) [97]	Australia	232	Pre and post-test	General emergency care	EM course	n/a	EM knowledge	Median SAQ-score (/10)	5 to 6 (p<0.001)	+	11
Rogers et al. (2000) [98]	USA	43	Non-randomised two-group	General emergency care	CCM elective	No elective	Ability to assess and manage a simulated acutely unwell patient	Mean OSCE checklist score (% points)	Increase by 26.9% (p<0.0001) in intervention group vs 10% in control group	++	14

These studies were conducted across nine regions, in which the USA was the most common (n=24, 33.3%), followed by Europe (n=13, 18.1%), and seven (9.7%) UK-based. The majority involved clinical year medical students (n=52, 72.2%), of which 35 (48.6%) were in their final year. The median number of study participants was 114 (IQR=95.5).

The majority of studies were single-group pre- and post-tests (n=39, 54.2%), with nine (12.5%) non-randomised and 24 (33.3%) randomised controlled trials (RCTs). The three most prevalent topic areas within acute care were trauma (n=16, 22.2%), followed by resuscitation (n=15, 20.5%), and general emergency care (n=14, 19.4%).

The majority of studies measured acute care knowledge (n=37, 51.4%), mostly using multiple-choice question (MCQ) tests (25/37) and short-answer question (SAQ) tests (7/37). Acute care assessment/management skills were evaluated in 23 studies (31.9%), 18 of which used checklist/global scoring methods, two measured specific outcomes (e.g. time to triage), and three used both approaches. The remaining 12 studies (16.7%) assessed student attitudes, mostly using Likert-scale questionnaires (10/12). No study evaluated behavioural changes or patient/healthcare outcomes.

The intervention types and their results

Six broad categories of interventions were identified: simulation, educational courses, clerkships, E-learning, peer-led teaching and curriculum modifications. The simulation studies have been further subdivided into “Simulation-only” and “Simulation with other teaching”. The studies incorporating courses have been further subdivided into “Courses without simulation” and “Courses with Simulation”.

The descriptions of the incorporated educational interventions and the findings at each outcome level are described in the subsections below. For each category and sub-category, the results of studies assessing student attitudes are presented first, followed by studies assessing acute care knowledge and subsequently acute care assessment/management skills.

Simulation

Simulation accounted for the majority of educational interventions (n=26, 36.1%). Six studies augmented simulation with one other teaching modality (e.g. E-learning) and were hence subtyped as “simulation with other teaching” [31, 40, 55, 67, 69, 78].

Simulation-Only

Seven studies measured student attitudes towards acute care. The majority of these (n=5) employed high-fidelity mannikin simulation (HFMS), which was defined as the use of computerised manikins to recreate scenarios with an enhanced level of realism, four of which were effective and one was highly effective [36, 63, 80, 85, 90, 99]. In particular, an RCT by Curran et al. improved student neonatal resuscitation Likert-scale confidence scores by 23.4% greater than the video-based learning comparator group [90]. Two studies used simulated live patients and produced 36% (p<0.01) and 2.9-fold (p<0.001) increases in self-reported confidence and preparedness scores, respectively [38,43].

Knowledge gains were assessed in five studies, though this was largely unsuccessful. Padaki et al. produced a 15.3% increase in MCQ-scores after using low-fidelity simulation (LFS), and two studies comparing HFMS against other educational strategies reported negative results [32]. An RCT by Gordon et al. found that lecture-based learning enhanced SAQ-scores by 27% more than HFMS, and a similar result with MCQ-scores was observed, when compared against case-based discussion (CBD) in a later non-randomised study [54,86]. One RCT, which incorporated HFMS, did, however, produce a 51% (p=0.0387) increase in MCQ-scores versus lecture-based learning and a pre- and post-test study, utilising simulated live patients, improved SAQ-scores by 36% (p<0.001) [45,47].

Of the eight studies evaluating acute care skills, six employed HFMS. An RCT by Steadman et al. reported a 2.1-fold (p<0.04) increase in overall checklist scores across nine simulated emergencies, versus problem-based learning (PBL) [83]. Three further pre- and post-test studies gave rise to effective and highly effective improvements [39,84,89]. Though two other RCTs produced variable effects, when HFMS was compared against CBD and video-based learning [71,94]. LFS was utilised in two studies, which yielded 84.7% (p<0.05) and 2.6-fold (p=0.0001) improvements in simulated acute care prescribing and emergency medicine (EM) skills checklist scores, respectively [46,57].

Simulation with Other Teaching

Berger et al. compared HFMS and PBL against classical resuscitation training, but an ineffective 7.7% difference in mean Likert-scale self-perception scores was observed [31].

Three studies measured knowledge acquisition using MCQ tests, of which two were successful. Thomson et al. combined LFS with E-learning and produced a 75% (p<0.001) increase in paediatric resuscitation knowledge, and a 35.6% increase in toxicology knowledge was observed when HFMS was augmented with PBL in Halm et al. [67,69]. However, an RCT by Cortegiani et al., which used HFMS alongside didactic teaching, produced an ineffective 14.2% mean improvement in acquisition of resuscitation knowledge, versus didactic-only teaching [55].

The remaining two studies, which assessed skills, augmented simulation with E-learning. Reed et al. utilised LFS and enhanced mean overall EM skill checklist scores by 82.2% (p<0.05), whilst Vincent et al. achieved >20% (p<0.001) increases in simulated triage scenario outcomes using virtual reality simulation [40,78].

Courses

Educational courses were the next most prevalent of interventions (n=25, 34.7%) and were defined as interventions consisting of multiple related teaching modalities. However, 10 studies included simulation within their courses and were thus subtyped as “courses with simulation.”

Courses Without Simulation

Student attitudes were assessed in three studies, in which two studies implemented the “Stop the Bleed” training course and yielded highly effective improvements in confidence towards haemorrhage management [27, 29]. Lin et al. described an emergency trauma training course, which improved mean trauma management Likert-scale confidence scores by 39% (p<0.001) [75].

All of the 10 studies which measured attainment of knowledge demonstrated effective (n=4) and highly effective (n=6) increases [30, 41, 44, 62, 87, 88, 91, 93, 97]. The Trauma Evaluation and Management (TEAM) course was implemented in six of these studies. A pre- and post-test study by Delgado-Reyes et al. improved trauma management MCQ-scores by 50% (p<0.01) [44]. The remaining five studies were non-randomised (n=1) and RCTs (n=4), all of which produced >50% increases in MCQ-scores with respect to the control groups (no TEAM course) [87, 88, 91, 92].

A non-randomised study by Ali et al. investigated the effects of the TEAM course on the acquisition of trauma assessment/management skills and reported >50% increases in checklist performance scores in a simulated scenario, versus the control group [95]. An RCT by Hansel et al. compared the use of a crew resource management course against simulator training and no training, in enhancement of situational awareness skills and performance in a simulated sepsis scenario, but produced variable results [66].

Courses with Simulation

Seven studies measured knowledge, of which four were effective [33, 51, 74, 77]. Three studies incorporated simulation within the TEAM course. A pre- and post-test study by Hill et al. increased trauma management MCQ-scores by 26% (p<0.001) [33]. When compared against the classroom-based TEAM course, an RCT by Ali et al., which utilised HFMS and simulated live patients, increased MCQ-scores by >20% versus the classroom-based TEAM course (p<0.05) [77]. An earlier RCT, which utilised simulated live patients, yielded a >50% improvement in knowledge versus the control group (no TEAM course), but failed to do so against the classroom-based TEAM course (13.2%) [82]. Two further studies generated ineffective gains in resuscitation SAQ-scores (14%) and trauma management MCQ-scores (16.5%) [49,59].

Three pre- and post-test studies assessed skills. Langdorf et al. implemented a “Resuscitation Boot Camp” and Ahmadi et al. conducted an advanced trauma life-support course, both of which improved simulated scenario performance scores by >50% (p≤0.03) [58,64]. Cuisinier et al. delivered a major trauma course, which improved median trauma assessment/management checklist scores by >20% (p<0.02) [53].

Clerkships

Eight studies (11.1%) either employed or modified existing clerkships. The mean duration was 3.7 weeks (range: 2-4). Merlin et al. delivered a four-week EM clerkship, but failed to yield effective increases in self-reported understanding of the triage system (8.1%) and comfort in using a defibrillator (10.8%) [73].

Two studies measured EM knowledge. A pre- and post-test by Afzalimoghaddam et al. increased student MCQ-scores by 80.3% (p<0.0001) following attendance of a four-week EM clerkship [70]. However, a non-randomised study by Lampe et al., which administered a standardised EM clerkship, was unable to produce an effective increase in MCQ-scores versus a regular clerkship (13.4%) [79].

The majority of these studies (5/8) evaluated skills, all of which were effective (n=1) and highly effective (n=4). Two studies implemented a one-month critical care medicine (CCM) elective [96,98]. A non-randomised study by Rogers et al. increased the mean simulated evaluation of a critically ill patient’s Observed Structured Clinical Examination (OSCE) scores by 1.7-fold greater than the control group (no elective) [98]. A later pre- and post-test produced a 61.7% increase in mean OSCE scores and enhanced performance on a computer simulator by 51.2% (p<0.0001) [96]. Two RCTs implemented Resident as Teacher Preceptorships during clerkships.28,48 Smith et al. improved simulated emergency management skills by 39.5% versus a regular clerkship, and this was by 100% (p=0.026) in Ruest et al. [28,48]. A non-randomised study by Woods et al. which provided students with a pocket-card and teaching on the resuscitation, analgesia and management, patient needs, interventions and disposition approach prior to undertaking an EM clerkship, demonstrated a 6.3-fold (p=0.006) greater increase in verbalised assessment of an acutely ill patient than a regular clerkship [56].

E-Learning

E-learning was described in six studies (8.3%). Four studies measured knowledge acquisition and reported mixed results. Two RCTs produced effective and highly effective increases in knowledge on adult resuscitation (31% and 28.1%) and paediatric emergencies (14.9-fold) relative to the control groups [60,65]. However, another RCT recorded a 12.1% (p>0.05) greater uptake of acute burns management knowledge than lecture-based learning [34]. Furthermore, lecture-based learning outperformed E-learning by 1.9-fold (p=0.0001) in a non-randomised study by Jordan et al. [61].

A pre and post-test study by O’Leary and Janson enhanced paediatric basic life-support (BLS) and advanced life-support (ALS) performance by 1.6-fold (p<0.001, 95%CI= 34.9-80.5) and 80% (p<0.001, 95%CI= 61.8-99.8), respectively [72]. However, when compared against standard paediatric BLS training in an RCT by Lehmann et al., blended learning was outperformed by >20% in observed paediatric resuscitation performance scores [52].

Peer-Led Teaching

Peer-to-peer teaching amongst medical students was undertaken in three studies (4.2%), all of which measured knowledge. Pean et al. described a peer-led EM skills course, which produced an 88.9% (p<0.05) increase in MCQ scores [50]. However, an RCT by Abbas et al., which investigated the improvements in MCQ scores between a peer-led and professional-led BLS course, was ineffective [35]. A non-randomised study by House et al., which compared peer-led against professional-led simulation for enhancing knowledge on cardiac emergencies, was also ineffective (p=0.6) [37].

Curriculum Modifications

Curriculum modifications were defined as interventions which implemented changes to previously existing curricula and did not fit into any of the above groups. Four studies (5.6%) were classed as such. Two studies integrated CBD within their curricula. Jordan et al. recorded a 3.1-fold (p<0.001) greater increase in EM knowledge when compared against self-directed learning [42]. However, a variable increase in BLS knowledge was observed when compared against traditional multimedia presentation [76]. Breckwoldt et al. trained medical students to teach BLS in schools, though this intervention performed negatively against the comparator groups with respect to BLS knowledge acquisition [81].

Nicol et al. implemented a vertically integrated resuscitation training curriculum, which improved observed BLS performance by 77.1% (p<0.05) [68].

Intervention Effectiveness

The effect-measures and MERSQI scores of each intervention type are summarised in Table 3. Of the 72 studies included in this review, 72.2% (95%CI=61.9-82.6) were classed as effective (n=22) and highly effective (n=30). Educational courses consisted of the highest proportion of effective and highly effective studies (84%, 95%CI=69.6-98.4), followed by clerkships (75%, 95%CI=45.0-105.0) and simulation (73.1%, 95%CI=56.0-90.1).

**Table 3:** Summary of effect-measures by intervention type and outcome level The effect-measures assigned to each intervention type (displayed in bold) are presented along with their mean (±SD) MERSQI scores. Beneath each intervention type is a breakdown of the effect-measures by outcome-level. To the right, the proportion of effective and highly effective studies overall and by each outcome-level is stated with their respective 95% CIs. At the bottom of the table, the total number of each effect-measure assigned is presented and beneath, the number of each effect-measure per outcome-level.

Intervention Type	n						++ or + (%)	95% CI (% + or ++)	MERSQI Scores	
	++	+	O	-	V	Total			Mean (±SD)	Range
Simulation	9	10	3	2	2	26	73.1	56.0-90.1	11.8 (±1.7)	7.8-15.5
Attitudes	2	5	1	0	0	8	87.5	64.8-110.4		
Knowledge	2	2	2	2	0	8	50	15.4-84.6		
Skills	5	3	0	0	2	10	80	55.2-104.8		
Courses	11	10	2	0	2	25	84	69.6-98.4	12.4 (±1.6)	8.4-15.5
Attitudes	2	1	0	0	0	3	100			
Knowledge	6	8	2	0	1	17	82.4	64.2-100.5		
Skills	3	1	0	0	1	5	80	44.9-115.1		
Clerkships	5	1	2	0	0	8	75	45.0-105.0	12.8 (±1.5)	10.2-14.5
Attitudes	0	0	1	0	0	1	0			
Knowledge	1	0	1	0	0	2	50	-19.3-119.3		
Skills	4	1	0	0	0	5	100			
E-learning	2	1	1	2	0	6	50	10-90	14.25 (±0.7)	13.5-15.5
Attitudes										
Knowledge	1	1	1	1	0	4	50	1.0-99.0		
Skills	1	0	0	1	0	2	50	-19.3-119.3		
Peer-led teaching	1	0	2	0	0	3	33.3	-20-86.7	12.3 (±2)	10.5-14.5
Attitudes										
Knowledge	1	0	2	0	0	0	33.3	-20.0-86.7		
Skills										
Curriculum modifications	2	0	0	1	1	4	50	1.0-99.0	13.4 (±1.3)	12-14.5
Attitudes										
Knowledge	1	0	0	1	1	3	33.3	-20.0-86.7		
Skills	1	0	0	0	0	1	100			
Total	30	22	10	5	5	72	72.2	61.9-82.6	12.5 (±1.7)	7.8-15.5
Attitudes	4	6	2	0	0	12	83.3	62.2-104.4		
Knowledge	12	11	8	4	2	37	62.2	46.5-77.8		
Skills	14	5	0	1	3	23	82.6	67.1-98.1		

Clerkships gave rise to the greatest proportion of effective and highly effective improvements in acute care skills (100%), followed by simulation (80%, 95%CI=55.2-104.8) and courses (80%, 95%CI=44.9-115.1).

Study Quality

The MERSQI domain scores are displayed in Table 4. The mean overall MERSQI score was 12.4 (range=7.8-15.5, SD=1.7), in which 18 (25%) studies had scores of ≥14. The lowest mean(±SD) domain scores were validity of the evaluation instrument (1.4±1.0), outcomes (1.4±0.2) and sampling (2.0±0.7). The median response rate was 95.2% (IQR=30.0); however, the majority of studies were single-centred (n=65, 90.3%) and only eight out of 61 applicable studies reported full validity evidence.

**Table 4:** MERSQI domain scores MERSQI: Medical Education Research Study Quality Instrument *Total achievable score for each domain is 3/3 ^Determined across 61 applicable studies

MERSQI Domain*	Mean Score	% Mean Score	SD
Study design	2.1	70	0.7
Sampling	2.0	66.7	0.3
Type of data	2.7	90	0.8
Validity of the evaluation instrument^	1.4	46.7	1.0
Data analysis	2.9	96.7	0.5
Outcomes	1.4	46.7	0.2

One-way ANOVA testing revealed a significant difference across the MERSQI scores of the intervention types (p=0.03). Post-hoc unpaired T-testing, however, only revealed significant differences between E-learning and simulation (p<0.0001, 95% CI=2.1-2.7), and E-learning and courses (p=0.00028, 95% CI=1.0-2.8).

Discussion

This review included a large body of literature, thereby indicating that a great deal of research has been conducted in this area. This is unsurprising, given the need to improve undergraduate preparation for acute care.

Quality of the Included Studies

There were few significant differences between the methodological quality of the intervention types; however, only 19 studies (26.4%) were of high-quality. Few studies were multi-institutional, the majority did not include control/comparator groups, reporting of validity evidence was poor and most importantly, no study measured behavioural changes or patient/healthcare outcomes. Whilst these outcomes are difficult to measure, they are paramount, as the ultimate purpose of this research is to enhance actual preparedness for acute care, such that patient outcomes are improved. A number of studies did measure improvements in skills, most of which were successful (19/23); however, this outcome represents the “shows-how” level of Miller’s pyramid [18]. This is, therefore, an estimate of future clinical performance rather than an actual indicator. Furthermore, only three studies directly compared the intervention types; hence, their comparative effectiveness was estimated based on the proportion of effective and highly effective studies [66, 84, 90]. These inherent limitations make it difficult to propose valid conclusions and recommendations, with which to influence curriculum reform. These findings can, however, be used to inform future research directions.

Effectiveness by Intervention Type

This review identified six types of educational interventions, of which courses, clerkships and simulation were found to be the most effective. The previous review by Smith et al. also described these interventions as “potential solutions” towards improving undergraduate acute care training, but did not evaluate their effectiveness relative to the other interventions presented in that review [11]. This review, to an extent, supports the implementation of these three interventions over others. Connell et al. also systematically reviewed the effectiveness of educational interventions on the recognition and management of deteriorating patients [100]. Whilst the use of simulation was supported, there was little mention of courses and none of clerkships, yet the review was conducted on healthcare professionals and included considerably fewer studies (n=23). This may account for these discrepancies in findings.

Although E-learning and peer-led teaching have also been identified as potentially useful acute care educational interventions, they were not found to be particularly effective in this review [10, 11]. Nevertheless, they consisted of few studies, and there is evidence of their success in other areas of medical education [101, 102]. Thus, further enquiry into their effectiveness may prove fruitful.

With regards to curriculum modifications, the literature has additionally encouraged the development of longitudinal, integrated curricula in acute care [10, 11], though only one study in this review implemented such an intervention [68]. Whilst a highly effective increase in BLS skills was observed, this study was limited by the fact that it was single-centred, did not involve a control/comparator group and reported a high rate of participant attrition (77.9%). Therefore, higher-quality studies assessing the use of this intervention are needed.

Improving Acute Care Skills

Whilst educational courses yielded the greatest proportion of effective and highly effective improvements in outcomes, only five of the 25 studies measured acute care skills. Moreover, subgroup-analysis, revealed that all of the “courses with simulation” studies (3/3) assessing skills produced positive findings, versus one out of two for “courses without simulation” [53,58,64,95]. Smith et al. recommended the inclusion of simulation within courses to improve acute care skills, and this was supported by a further systematic review [11,103]. Hence, courses with simulation may amplify the acquisition of acute care skills, though further research is needed, given the limited number of such studies in this review.

In contrast, the majority of clerkship studies (5/8) evaluated the acquisition of acute care skills, all of which reported successful findings. Smith et al. reasoned that clerkships improve practical skills through providing “hands-on experience” [11]. A literature review on undergraduate critical care education issued further evidence to that effect, and a large multicentred qualitative study conveyed the importance of students’ “learning on the job” [9,104]. This finding, additionally, reflects the superiority of authentic experiences versus the artificiality of other learning methods, as evidenced in a questionnaire study by Burford et al., which demonstrated a greater effect of “real-life” acute care experiences on self-perceived preparedness than with simulation [105]. However, the considerably smaller number of clerkship studies compared to simulation and courses may have confounded these results. This comparative lack of studies suggests a paucity of research into this intervention, and a subsequent need for more.

Simulation also improved skills in the majority of studies, and there is a myriad of evidence to support its effectiveness in the acquisition of acute care skills [10, 106, 107]. The literature additionally favours HFMS over LFS; however, this review contained far fewer LFS studies (n=5) to enable a fair comparison with HFMS (n=17), and none directly compared both approaches [107, 108]. The use of simulated live patients also appeared promising, but there were too few studies (n=3) to draw meaningful conclusions. Nevertheless, simulation alone did not appear to enhance acute care knowledge, especially when compared against other teaching modalities [54, 86]. This is evidenced further by two meta-analyses, which found simulation to be ineffective versus non-simulation modalities [107, 109]. Although this outcome lies at the bottom of Miller’s hierarchy, it is still important, given the assumption that one needs to possess the correct knowledge in order to competently assess/manage acutely unwell patients [18]. Beal et al. proposed augmenting simulation with other teaching methods, which supports the notion that integration with other curricular activities amplifies its effectiveness [107, 110, 111]. In this review, two out of three of the “simulation with other teaching” studies improved knowledge [67, 69], though this was only the case in four out of seven of the “courses with simulation” studies [33, 51, 74, 77]. This warrants further investigation into the effects of simulation with one or more educational modalities.

Strengths and limitations

The strengths of this review lie in the systematic search strategy, rigorous selection criteria and the substantial efforts made to synthesise the effectiveness of a large body of literature in a manner that was understandable, comparable and reproducible.

This review was firstly limited by the considerable heterogeneity of the designs, topic areas and measured outcomes of the included studies. This not only prevented meta-analysis but also the undertaking of an in-depth narrative synthesis. A more focused area of review may have proved more beneficial.

Secondly, this review was subject to publication bias, as only peer-reviewed literature was included, and a grey-literature search was not conducted. Therefore, potentially useful studies may have been missed. Additionally, the majority of abstracts and full-texts were screened by one reviewer, as well as the fact that there is a dearth of included literature after 2020. These serve as further limitations of this review.

Thirdly, there was a mismatch between the effect-measures assigned and reported p-values in eight studies (11.1%) [28, 32, 49, 73, 79, 82, 86, 94]. This meant that some studies were classified as ineffective, though their p-values indicated statistical significance and vice versa. This undermines the construct validity of this approach because it is unclear whether the changes seen in outcomes were due to the intervention or chance alone. It would have been ideal to measure effectiveness based on the reported p-values, as previously employed by Gill et al.; however, 22 studies (30.6%) did not report the correct p-values to permit that approach [20]. This method, therefore, served as an alternative.

Furthermore, this review did not evaluate the rates of retention/attrition of the outcomes measured over time. Consequently, the long-term effects of these interventions, which are more likely to translate into clinical practice, remain unknown.

Lastly, the cost-effectiveness of the interventions was not considered in this review, and this should be evaluated in further studies. Institutions possess a finite amount of money and resources; therefore, it is important to take into account the effectiveness of educational interventions within the financial constraints of curriculum design.

## Conclusions

This review aimed to build upon the findings of previous reviews by formally evaluating the effectiveness of different educational interventions. These findings particularly favour the use of clerkships, as well as courses and simulation, to enhance medical student preparation for acute care. However, considerable heterogeneity and the presence of several limitations at study and outcome-level, especially the absence of data on changes in student behaviour or patient/healthcare outcomes, prevent any definitive conclusions from being drawn.

As a consequence, optimisation of the quality of future studies by measuring such outcomes, ideally being multicentred, including control/comparator groups and reporting full applicable evidence of validity, is needed to confirm the true effectiveness of these interventions. Additional avenues for future research include further investigation into the effectiveness of integrated curricula and the use of simulation within courses or with other teaching modalities. Further reviews assessing long-term outcomes and cost-effectiveness of interventions would also prove an invaluable contribution to the existing body of literature.

## Appendices

Complete search strategy for each database

**Table 5:** Complete search strategy for each database

MEDLINE and CENTRAL
1. Education, Medical, Undergraduate/
2. "medical student*".ti,ab.
3. Emergency Medicine/
4. Critical Care/
5. Traumatology/
6. "acute care".ti,ab.
7. "immediate care".ti,ab.
8. acute care surg*.ti,ab.
9. Education, Medical/
10. exp Curriculum/
11. exp Teaching/
12. improv*.ti,ab.
13. train*.ti,ab.
14. exp Professional Competence/
15. Educational Measurement/
16. Health Knowledge, Attitudes, Practice/
17. "prepar* for practic*".ti,ab.
18. 1 or 2
19. 3 or 4 or 5 or 6 or 7 or 8
20. 9 or 10 or 11 or 12 or 13
21. 14 or 15 or 16 or 17
22. 18 and 19 and 20 and 21
Embase
1. medical student/
2. "medical stud*".ti,ab.
3. emergency medicine/
4. exp emergency care/
5. "emergency car*".ti,ab.
6. "acute car*".ti,ab.
7. "immediate car*".ti,ab.
8. acute care surg*.ti,ab.
9. exp medical education/
10. curriculum/
11. curriculum development/
12. education program/
13. teaching/
14. improv*.ti,ab.
15. train*.ti,ab.
16. clinical competence/ or professional competence/
17. professional knowledge/
18. clinical practice/
19. skill retention/
20. "prepar* for practic*".ti,ab.
21. 1 or 2
22. 3 or 4 or 5 or 6 or 7 or 8
23. 9 or 10 or 11 or 12 or 13 or 14 or 15
24. 16 or 17 or 18 or 19 or 20
25. 21 and 22 and 23 and 24
Scopus
(TITLE-ABS-KEY("medical stud*") ) AND (TITLE-ABS-KEY(emergency medicine) or TITLE-ABSKEY(emergency car*) OR TITLE-ABS-KEY(acute medicine) OR TITLE-ABS-KEY(acute car*) OR TITLE-ABS-KEY(critical car*) OR TITLE-ABS-KEY(immediate car*) OR TITLE-ABS-KEY(acute care surg*) OR KEY(trauma) ) AND (KEY(medical education) OR KEY(curriculum) OR TITLE-ABSKEY(teach*) OR TITLE-ABS-KEY(train*) OR TITLE-ABS-KEY(improv*) OR TITLE-ABSKEY(prepar*) ) AND (TITLE-ABS-KEY("prepar* for practic*") OR KEY(competence) OR KEY(professional knowledge) ) AND (NOT INDEX(medline) ) AND (PUBYEAR > 1999)
Web of Science
(TS="medical stud*") AND (TS= (emergency and medicine) OR TS=(emergency and car*) OR TS=(acute and medicine) OR TS=(acute and car*) OR TS= (immediate and car*) OR TS=(critical and car*) OR TS= (acute and care and surg*) OR TS=(trauma) ) AND (TS=(medicaland education) OR TS=(curriculum) OR TS=(teach*) OR TS= (train*) OR TS=(improv*) OR TS=(prepar*) ) AND (TS=("prepar* for practic*") OR TS=(competence) OR TS=(professionaland knowledge) )
